# Comparación de la escala qSOFA para evaluar la falla orgánica secuencial y los criterios SIRS para sepsis a la cabecera de pacientes con bacteriemia por *Staphylococcus aureus*

**DOI:** 10.7705/biomedica.4943

**Published:** 2020-08-20

**Authors:** Óscar García, Tatiana Álvarez, Santiago Granados, Vanessa Garzón, Santiago González

**Affiliations:** 1 Grupo de Investigación en Medicina interna, Universidad Tecnológica de Pereira, Pereira, Colombia Universidad Tecnológica de Pereira Grupo de Investigación en Medicina interna Universidad Tecnológica de Pereira Pereira Colombia

**Keywords:** Staphylococcus aureus, *Staphylococcus aureus* resistente a meticilina, bacteriemia, infección hospitalaria, puntuaciones en la disfunción de órganos, síndrome de reacción inflamatoria sistémica, Staphylococcus aureus, methicillin-resistant *Staphylococcus aureus*, bacteremia, cross infection, organ dysfunction scores, systemic inflammatory response syndrome

## Abstract

**Introducción:**

*Staphylococcus aureus* es una de las principales causas de bacteriemia, adquirida en la comunidad o asociada con la atención en salud, la cual presenta un gran porcentaje de complicaciones y elevadas tasas de morbilidad y mortalidad. Los criterios SRIS (*Systemic Inflammatory Response Syndrome*) se han usado tradicionalmente con el fin de establecer la presencia de sepsis; sin embargo, recientemente se ha cuestionado su valor predictivo dada su baja sensibilidad y especificidad. En el 2016, apareció la escala qSOFA (*quick Sequential Organ Failure Assessment*), como una nueva herramienta para la evaluación rápida de las infecciones en los servicios de urgencias.

**Objetivo.:**

Comparar las herramientas qSOFA y SRIS para la predicción de la bacteriemia por *S. aureus*.

**Materiales y métodos.:**

Se hizo un estudio observacional sobre el comportamiento clínico de pacientes con bacteriemia por *S. aureus* para evaluar el perfil de resistencia fenotípica, algunas características sociodemográficas, clínicas y de laboratorio, las complicaciones y la mortalidad, así como los resultados de las evaluaciones con la escala qSOFA y los criterios SRIS, para establecer cuál podría predecir mejor la presencia de bacteriemia por *S. aureus*.

**Resultados.:**

Se seleccionaron 26 pacientes con bacteriemia, en cuyas muestras *S. aureus* había sido el segundo germen más frecuentemente aislado. Se encontró una mortalidad del 50 % (13 casos) y una prevalencia del 30 % de *S. aureus* resistente a meticilina (SARM). Según los puntajes clínicos obtenidos, la escala qSOFA fue positiva en 30,8 % de los pacientes y los criterios SRIS lo fueron en el 92,3 %.

**Discusión.:**

Se encontró una elevada mortalidad en la población analizada. La escala qSOFA fue menos efectiva para el diagnóstico que los criterios clásicos de reacción inflamatoria sistémica.

## Abstract (en)

**Introduction:**

One of the primary causes of community-acquired bacteremia also associated with nosocomial infections is *Staphylococcus aureus,* which is responsible for a high percentage of complications leading to high morbidity and mortality rates. The systemic inflammatory response syndrome (SIRS) criteria have been traditionally used to evaluate the presence of sepsis; however, recent evidence questions its predictive value due to its low sensitivity and specificity. In 2016, new criteria for sepsis were published and a new tool emerged, quick SOFA (qSOFA), for the rapid evaluation of infection in emergency services.

**Objective:**

To compare the qSOFA and SRIS tools for the prediction of bacteremia caused by *S. aureus*.

**Materials and methods:**

We conducted an observational study in patients with *S. aureus* bacteremia to evaluate their phenotypic resistance patterns, some special features (sociodemographic characteristics, clinical and paraclinical values), complications, and mortality. The results of the qSOFA and SIRS scales were analyzed to identify which of them could better predict the presence of *S. aureus* bacteremia.

**Results:**

Twenty-six bacteremic patients were identified. *Staphylococcus aureus* was the second most frequently isolated bacteria. The results evidenced a mortality rate of 50% (13 cases) and a prevalence of 30% of MRSA. For the clinical scores evaluated, the qSOFA scale was positive in 30.8% of the patients, and the SIRS scale, in 92.3%.

**Discussion:**

The mortality rate for the population under study was high and the qSOFA tool had a lower diagnostic yield compared to the classic criteria for SIRS.

Los criterios diagnósticos para la tamización de los pacientes con sepsis fueron modificados ante la publicación de un estudio de seguimiento epidemiológico a partir de bases de datos de Australia y Nueva Zelanda, en el cual se demostraba que, cuando se cumplían dos de los cuatro criterios clásicos del síndrome de reacción inflamatoria sistémica (SRIS) ^1^, se excluía a uno de cada ocho pacientes con infección, falla orgánica y riesgo sustancial de muerte ^2^.

Dicha comunicación obligó a modificar la actitud hacia este síndrome, que constituye un problema de salud pública a nivel mundial ^3^. El inicio del tratamiento antibiótico afecta la morbimortalidad del paciente infectado ^4^, lo que exige optimizar la oportunidad del diagnóstico. Esta premisa fue la base para que, en un estudio posterior en el que se utilizaron modelos de regresión logística con información bayesiana, se plantearan nuevos criterios para detectar a los pacientes con cuadros infecciosos. Dicho modelo dio origen a la herramienta conocida como escala qSOFA (*quick Sequential Organ Failure Assessment*) ^5^, una herramienta clínica sencilla, especialmente útil en los servicios de urgencias pues, al comparar el área bajo la curva con otros puntajes de tamización, fue el que estuvo más cercano a 1 (0,81: 0,80-0,82), resultado que no se obtuvo en pacientes infectados en el servicio de cuidados intensivos, en donde el área bajo la curva fue de apenas 0,66 (0,64-0,68).

*Staphylococcus aureus* es un importante germen patógeno en hospitales y en la comunidad ^6^, y una de las principales fuentes de bacteriemia ^7^, la cual se cuenta entre las condiciones patológicas más graves debido a su morbimortalidad. La tasa de mortalidad asociada con la bacteriemia por *S. aureus* puede alcanzar el 50 % ^8^.

Para la tamización no existe una prueba de referencia que compruebe la presencia de infección, pues son múltiples las enfermedades que simulan la sepsis ^9^. En este contexto, se evaluaron dos herramientas para detectarla a la cabecera de los pacientes.

## Materiales y métodos

Se llevó a cabo un estudio observacional de corte transversal. Cada dos días se recolectó información sobre todos los hemocultivos procedentes de los servicios de medicina interna, urgencias y cuidados intensivos, entre el 1° de junio de 2017 y el 1° de diciembre de 2017, en una institución de salud pública de tercer nivel en Pereira.

Se seleccionaron los hemocultivos positivos para *S. aureus* y se hizo el seguimiento clínico a la cabecera de los pacientes, recolectando los datos sobre las variables clínicas en un formato que incluía la escala qSOFA y los criterios SRIS. Se evaluaron, asimismo, las características sociodemográficas, los factores de riesgo, las complicaciones y los resultados finales.

El análisis estadístico se hizo con el programa Stata™, la detección microbiológica, con el sistema VITEK 2™ (bioMérieux), y el análisis microbiológico, con WHONET 5.6.

El estudio obtuvo el aval ético del Comité de Ética de la Universidad Tecnológica de Pereira (Acta No. 31, punto 03, numeral 3.2.1, del 17 de abril de 2017) y del Comité de Ética del Hospital Universitario San Jorge (radicado No 2017003015 del 18 de mayo de 2017).

## Resultados

De los 164 pacientes con hemocultivos positivos en el periodo estudiado, 26 (15,8 %) lo fueron para *S. aureus* y, de este porcentaje, el 96 % tuvo más de un hemocultivo positivo para dicho microorganismo*.* Las características demográficas y clínicas de los pacientes se presentan en el cuadro 1. El tratamiento empírico más frecuentemente utilizado fue la combinación de vancomicina y oxacilina (23 %). Otros antibióticos menos usados (7,7 % cada uno) fueron piperacilina-tazobactam, cefepime, cefepime-vancomicina y meropenem-vancomicina. El esquema de tratamiento se ajustó según el antibiograma, en todos los pacientes. En cuanto a las puntuaciones clínicas, el 30,8 % de los pacientes tuvieron un puntaje positivo en la qSOFA, y el 92,3 % cumplieron con dos o más de los criterios SRIS.

**Cuadro 1:** Características demográficas y clínicas de los pacientes con infección por *Staphylococcus aureus* (N=26)

Característica			
Sexo, n (%)	Hombres: 11 (42,3)	Mujeres: 15 (57,7)
Edad (años)	58,1	Rango: (20-85)
	Hombres: media: 56,2	Rango: (30-76)
	Mujeres: media: 59,5	Rango: (20-85)
Procedencia, n (%)	Urbana: 18 (69,2)	Rural: 8 (30,8)
	Hombres: 9	Hombres: 2
	Mujeres: 9	Mujeres: 6
Régimen de afiliación al SGSSS, n (%)	Subsidiado o vinculado: 23 (88,4)	Otros: 3 (11,6)
Estancia hospitalaria (días)	Promedio: 34,3	Rango: (5-70)
Uso previo de antibióticos, n (%)	Sí: 10 (38,5)	No: 16 (61,5)
Presencia de catéter central, n (%)	Sí: 11 (42,3)	No: 15 (57,7)
Estancia en sitios de cuidado de la salud,	Sí: 16 (61,5)	No: 10 (38,5)
n (%)		
Historia de diabetes mellitus, n (%)	Sí: 11 (42,3)	No: 15 (57,7)
Historia de enfermedad renal crónica ≥3,	Sí: 13 (50)	No: 13 (50)
n (%)		
Infección asociada con el cuidado de la	Sí: 14 (53,8)	No: 12 (42,2)
salud, n (%)		
Puntuación en la qSOFA, n (%)	Positivo: 8 (30,8)	Negativo: 18 (69,2)
Puntuación en los SRIS, n (%)	Positivo: 24 (92,3)	Negativo: 2 (7,7)
Tratamiento antibiótico (días)	Promedio: 17,2	DE: 15,7
Complicaciones al egreso	Presentes: 11 (42,3)	Ausentes: 15 (57,7)
Tipo de *Staphylococcus aureus*	SARM: 8 (30)	SASM: 18 (70)

SGSSS: Sistema General de Seguridad Social en Salud; DE: desviación estándar; SARM: *Staphylococcus aureus* resistente a meticilina; SASM: *Staphylococcus aureus* sensible a meticilina

La prevalencia de *S. aureus* resistente a la meticilina (SARM) fue del 30,7 % (8 casos). No se encontró relación significativa entre haber recibido antibióticos previamente o presentar una infección asociada con la atención en salud, y la presencia de resistencia a la meticilina (p=0,648 y p=0,683, respectivamente). En este grupo de pacientes, no se incrementó la mortalidad ni se prolongó la hospitalización frente a aquellos con *S. aureus* sensible a la meticilina (SASM).

El 50 % de los pacientes tenía enfermedad renal crónica en estadio 3 o mayor y la hemodiálisis fue la terapia de reemplazo renal más frecuente. El 42 % de los pacientes (11 casos) tenía catéter central en el momento de la toma del hemocultivo y en tres de ellos se aisló SARM, aunque no se encontró una relación significativa (p=1,0).

La mortalidad general fue del 50 %. En el grupo de pacientes con SARM, la mortalidad fue del 50 % y el 75 % había adquirido la infección en la comunidad. En el caso de SASM, la mortalidad fue también del 50 % y el 66 % lo había adquirido en la comunidad.

Se encontró una relación significativa entre la mortalidad y la infección asociada con la atención en salud (75 % de los casos mortales, p=0,047). No hubo diferencias significativas en cuanto a la mortalidad asociada con el sexo o el régimen de seguridad social; sin embargo, sí se encontró una diferencia estadísticamente significativa entre la procedencia urbana (92 % de los pacientes fallecidos) y la rural (p= 0,015).

El 73 % de la población estudiada tuvo tres hemocultivos positivos y, de este porcentaje, el 57,9 % murió, aunque la relación no fue significativa (p=0,378). Los resultados del análisis bivariado se presentan en el cuadro 2.

**Cuadro:** 2. Resultados del análisis bivariado

Variable	Sí	No	p
Mortalidad con aislamiento de SARM	4	4	0,78
Uso previo de antibióticos y aislamiento de SARM	3	5	0,648
Infección asociada con la atención en salud y	5	9	0,683
aislamiento de SARM			
Uso de catéter central y presencia de SARM	3	8	1,0
Mortalidad y procedencia urbana	7	1	0,015
*Mortalidad e infección comunitaria*	6	2	0,047

## Discusión

Los pacientes con bacteriemia por *S. aureus* pueden desarrollar una amplia variedad de complicaciones difíciles de reconocer, y que tienen impacto en la morbilidad y la mortalidad. Se han resgistrado tasas de mortalidad del 20 al 40 %, aparentemente mayores cuando se trata de infección con SARM comparada con la de SASM. En este estudio, se encontró una prevalencia de bacteriemia del 15 % y *S. aureus* fue el segundo germen más frecuentemente aislado.

Predominó el sexo femenino (57 %) al igual que en otras series publicadas ^10^, con excepción de otra serie ^11^ de más de 3.000 pacientes en cinco países y 20 centros, cuyo porcentaje de hombres fue del 64 %; no obstante, esta diferencia entre sexos no tuvo significación estadística en el análisis bivariado. El promedio de edad fue de 58 años, similar al de otras series (60 años) ^10^. En cuanto a la procedencia, el 69,2 % de los pacientes procedía del área urbana, hallazgo cuya significación se ve limitada por la distribución de la población en el departamento de Risaralda, ya que la mayoría reside en las ciudades. Además, el Hospital Universitario San Jorge es centro de referencia para todos los municipios de Risaralda, y para pacientes afiliados y vinculados al régimen subsidiado. La estancia hospitalaria fue mayor en los pacientes procedentes de los municipios de Risaralda (34 días en promedio), en comparación con lo registrado por otros autores (16 días en promedio) ^11^.

En cuanto a los factores de riesgo, se encontraron antecedentes de diabetes mellitus en 42 % de los casos y de enfermedad renal crónica en 50 %, prevalencias más altas que las de estudios con poblaciones más grandes (24,7 y 22 %, respectivamente) ^12^. Se resaltan estas diferencias porque son condiciones que comprometen la reacción inmunológica.

En cuanto a la efectividad de las escalas de puntuación para la tamización de los pacientes con sepsis, la qSOFA tuvo un bajo desempeño (resultado positivo en el 30,8 % de los pacientes), en contraste con el puntaje de los criterios SRIS, los cuales presentaron una gran sensibilidad (positivo en 92,3 % de los pacientes). En un metaanálisis de 10 estudios con 229.480 pacientes, se encontraron resultados similares, con una sensibilidad del 84,4 % para los criterios SRIS ^13^.

El presente estudio tiene la fortaleza adicional de que todos los pacientes tenían infección comprobada por hemocultivo mientras que, en el estudio poblacional mencionado, se incluyeron cultivos de múltiples muestras y se adoptó una definición propia de infección.

En un estudio retrospectivo reciente en 402 pacientes con bacteriemia por *S. aureus*, se encontró que el 98 % de ellos, con 2 o más puntos en la qSOFA, cumplía los criterios SRIS, en tanto que el 74 %, con 2 o menos puntos en esta escala, cumplían con dichos criterios, con una diferencia estadísticamente significativa (p<0,0001), lo cual constituye un hallazgo similar al del presenre estudio ^14^.

Además, en un estudio en el que se evaluó el rendimiento de las mismas escalas de puntuación aquí analizadas, aunque orientadas a la predicción de mortalidad y no de infección, el área bajo la curva fue de 0,56 para los criterios SIRS, de 0,67 para la escala qSOFA y de 0,73 para la qSOFA sumada a la prueba de procalcitonina, resultados que no reflejan un apropiado rendimiento de la escala para dicho fin. Cabe mencionar que, en este estudio, el 13 % de los pacientes cursaba con bacteriemia, que de ellos solo el 6 % tenía bacteriemia por bacterias Gram positivas y que no había información sobre este subgrupo en cuanto a la predicción de infección en el momento del ingreso ^15^. En otro estudio de predicción de la mortalidad con la qSOFA en pacientes geriátricos con influenza, el área bajo la curva fue de 0,81 ^16^.

En cuanto al uso de tratamiento antibiótico empírico, la combinación más frecuente (23 %) fue la de vancomicina y oxacilina, pero no se pudo establecer si fue apropiada. En el caso del tratamiento antibiótico dirigido, todos los pacientes fueron manejados de forma apropiada según el patrón fenotípico identificado en el antibiograma. En otro estudio en una cohorte de pacientes en 10 hospitales (562 casos), se encontró que solo el 51,8 % de los individuos recibió el tratamiento empírico de forma apropiada ^17^; en el análisis multivariado se encontraron algunas variables predictivas del tratamiento antibiótico adecuado, entre ellas, estar en hemodiálisis (*odds ratio*, OR=1,36) y tener un catéter venoso central en el momento del ingreso (OR=1,72).

En cuanto a la presencia de complicaciones, se encontró una prevalencia de 42 %, que es un valor esperado dentro del rango reportado en la literatura especializada ^7^. En algunas series se reportan prevalencias bajas de complicaciones (9,4 %) ^12^, lo que debe ajustarse por otras características de la población. En otros estudios, menos de la mitad de los pacientes con complicaciones presentaba diabetes o enfermedad renal crónica. En el presente estudio, la mortalidad fue del 50 %, lo cual debe interpretarse teniendo en cuenta que la población evaluada vivía en condiciones socioeconómicas desfavorables, lo que usualmente implica que las comorbilidades no han sido tratadas adecuadamente.

En la presente serie, el 30 % de las cepas de *S. aureus* fueron resistentes a la meticilina, porcentaje menor al de algunas otras series (56,8 %) ^18^. A pesar de esto, en el análisis bivariado no se incrementó la mortalidad comparada con la de los pacientes con bacteriemia por SASM. Se estableció que el 53 % de los pacientes cursaba con una infección asociada con la atención en salud, pero sin que ello se asociara con la presencia de SARM (p=0,683). En otras series, se ha reportado una tasa de mortalidad por SARM mayor que la de aquella por SASM, con un RR de 1,7 (IC_95%_ 1,3-2,4; p<,01) ^19^.

Una fortaleza del presente estudio fue la rigurosidad del seguimiento a los pacientes, a quienes, una vez detectados, se les hacía una evaluación clínica hospitalaria y se les seguía hasta el egreso o el fallecimiento sin limitarse a la evaluación exclusiva de las historias clínicas.

Como limitación debe mencionarse el registro de la mortalidad, ya que, debido a las elevadas tasas, no pudo establecerse con precisión si la muerte se debía a la bacteriemia, para lo cual se hubiera requerido un ajuste de variables. El tamaño de la muestra no permitió hacer un análisis estadístico más extenso. No obstante, este es el primer estudio en la ciudad que caracteriza a los pacientes con esta condición, lo que constituye un punto de referencia para futuras investigaciones en otras instituciones de salud de la ciudad y de la región del Eje Cafetero.
